# Effects of the Targeted Intervention for Five- to Six-Year-Old Children Affected by Attentional and Concentration Developmental Risks: Results of a Dynamic Prospective Cohort Study Conducted in Socially Deprived Regions in Germany

**DOI:** 10.1007/s11121-022-01362-8

**Published:** 2022-03-30

**Authors:** Marco Franze, Josefin Biermann, Anika Kästner, Vanessa Sophie Ernst, Wolfgang Hoffmann

**Affiliations:** Institute for Community Medicine, Section Epidemiology of Health Care and Community Health, University Medicine Greifswald, Ellernholzstr. 1-2, 17487 Greifswald, Germany

**Keywords:** Attention, Concentration, ADHD, Children, Preschool, Developmental screening, Social inequality, Policy

## Abstract

Epidemiological data reveal that there is a need for prevention measures specifically targeted at children with low SES. In the German federal state Mecklenburg-Western Pomerania preschools in socially deprived regions can apply for additional funds to support children with developmental risks. Mandatory criteria for obtaining these funds involve an annual assessment of all children using the “Dortmunder Developmental Screening for Preschools (DESK 3–6 R).” This instrument can detect and monitor developmental risks in the domains fine motor skills, gross motor skills, language, cognition, and social development. In this study, we examine the domain “Attention and concentration,” which is included for the 5 to 6-year-old age group, using data from two consecutive survey waves (sw). Research questions: (1) Does the prevalence rate ratio (PRR) improve over time? (2) Is the rate of improvements (developmental risk at sw1, no developmental risk at sw2) higher than the rate of deteriorations (no developmental risk at sw1, developmental risk at sw2)? Prospective cohort analysis (*n* = 940). The prevalence rate of a developmental risk in this DESK domain decreases over time (PRR = 0.78; *p* = 0.019). The ratio of the rate of improvements is 8.47 times higher than the rate of deteriorations. The results provide evidence of the effectiveness of targeted intervention measures in preschools focusing on skills that improve attention and concentration. This is significant considering the small-time interval and the categorization method of DESK scores. Nevertheless, over the same time period, the DESK results of some children deteriorated. Therefore, preschools also have to be aware that it is natural for some children to show modest declines in their skills over time. German Clinical Trials Register, ID: DRKS00015134, Registered on 29 October 2018, retrospectively registered.

## Introduction

Attention-deficit/hyperactivity disorder (ADHD) is comprised of the three core symptoms: inattentiveness, hyperactivity (motor unrest), and impulsivity. Relevant diagnostic criteria are its persistence (at least 6 months),: developmentally inappropriateness, pervasiveness across settings (e.g., school and home), and association with substantial functional psychosocial impairment. ADHD affects 5–5.5% of children worldwide (Faraone et al., 2015) and is directly associated with school engagement (Nguyen et al., 2019), i.e., the student’s perceived feeling of investment and motivation in school life (Appleton et al., 2008). School engagement is a protective factor against emotional distress, low mood, and suicidal behavior (Millings et al., 2012; Resnick & Bearman, 1997). With regard to high school graduation, the drop-out-rate is much higher in children affected by ADHD (22.9%) than in non-ADHD controls (10.0%) (Barbaresi et al., 2007). Therefore, there is a need for measures to prevent ADHD.

Addressed to preschool-aged children, such measures include screenings conducted by primary care clinicians (Subcommittee on Attention-Deficit/Hyperactivity Disorder, 2011). This is supposed to occur if children are affected by academic or behavioral problems and demonstrate symptoms of inattention, hyperactivity, or impulsivity. Additionally, recommended strategies include parental training (Mulqueen et al., 2015) and programs that incorporate games and play-based strategies to enhance children’s self-regulation (Halperin et al., 2014; Plueck et al., 2015).

### Early Childhood Education and Care

One example of an effective preschool teacher-based intervention is the so-called “Good Behavior Game” which can be used within a universal prevention approach targeted at all children in the preschool group (Humphrey et al., 2021). Furthermore, selective prevention approaches of preschool teachers, e.g., those realized by the Chicago School Readiness Project model, which address children at risk, may also be effective to reduce internalizing and externalizing behavior problems (Raver et al., 2009).

Nevertheless, children’s educational and health status is tremendously affected by social inequalities. Children from families with low socioeconomic status (SES) are between 2.8 and 4.2 times more likely to be affected by mental health problems comprising hyperactivity, emotional problems, behavior problems, and peer problems than children from families with higher SES (Kuntz et al., 2018b). A low SES also affects social-emotional skills (Rudolph et al., 2013), phonological awareness (Begić et al., 2019), numeracy (Anders et al., 2013), and motor skills (Gottschling-Lang, 2013, 2016). Low SES is associated with poorer informal learning at home, resulting in children being less well prepared for formal schooling and at greater risk of poor health literacy (Hoff, 2006; Hoff & Tian, 2005). In Germany, a low SES is associated with less frequent participation in the early childhood health screening and monitoring program (Rattay et al., 2014). Moreover, children from families with low SES do engage in sports activities less frequently (58%) than children from families with high SES (83.1%) (Kuntz et al., 2018a). They are more likely to be overweight or obese than children from families with high SES (low SES: 66.4%, high SES: 87.1%) (Krause & Lampert, 2014; Kurth & Schaffrath Rosario, 2007; Manz et al., 2014). Therefore, there is a need for prevention measures especially addressed to children from families with low SES.

In 2011, the government of MWP passed the federal state law for child day care and preschools (Ministry of Social Affairs, 2020). This law aims to foster children’s healthy development and to reduce social inequalities, leading to two promotion strategies: a promotion integrated into the daily routine and targeted at all children vs. a targeted individual promotion addressed to children affected by a developmental risk. Providers of preschools in socioeconomically deprived regions obtain an extra budget of EUR 5 million annually, which is mainly used to cover the personnel costs incurred in the implementation of a targeted intervention. The eligibility criterion for preschool providers is the amount of fees that are subsidized by the youth welfare offices rather than being paid by the parents. If the preschool-specific proportion of subsidized fees is above the mean, then the youth welfare offices inform providers of selected preschools about the opportunity to obtain additional grants and benefits (annual amount between EUR 10,000 and EUR 55,000 per preschool). To claim the funds, preschools must commit to annually conduct a valid developmental screening for all 3- to 6-year-olds (DESK 3–6 R; see “Instrument”) and to subsequently conduct a targeted individual promotion targeted at children affected by a developmental risk. Since this law does not clearly set out how preschools should conceptualize this targeted individual promotion preschools are free to develop measures on their own and to include further partners, e.g., speech and language therapists, occupational therapists, or physiotherapists (see also “Implementation of the Study”). These “DESK preschools” also have to participate in an evaluation of the effects of the federal state law (Franze et al., 2018). The assessments include annual DESK data from all participating preschools. The DESK data are individually linked so that trajectories can be longitudinally analyzed on a pseudonymized child-specific level.

This law is to be welcomed, for preschool attendance fosters children’s health and educational status (Brown et al., 2018; D'Onise et al., 2010; Sierens et al., 2020). This is crucial since social-emotional skills, early literacy, numeracy, and motor skills are associated with later educational and health outcomes (Denham, 2006; Hendrix et al., 2014; Kucian & von Aster, 2015; Logan et al., 2012; Navsaria & Sanders, 2015). Social-emotional skills, early educational achievements, and motor skills are relevant for good mental and physical health, attaining a college degree, and earning a high school diploma (Jones et al., 2015). However, the impact of early childhood education and care on children’s competencies depends on the professional development of the preschool staff (Jensen & Rasmussen, 2019), the quality of pedagogic interactions between preschool staff and children (Ulferts et al., 2019), and an appropriate preschool staffing ratio, i.e., in the case of 3- to 6-year-olds a ratio of 1:7.5 (Bock-Famulla et al., 2020). Yet, the actual ratio varies greatly among the German federal states. In 2018, in Mecklenburg-Western Pomerania (MWP), the median of the preschool staffing ratio was 1:13.2 which was the highest in Germany. This is challenging, at the very least, for the establishment of high-quality interactions between children and preschool teachers.

### Research Question

Keeping both the need for prevention measures for children with low SES and the preschool staffing ratio in MWP in mind, the question arises as to whether the targeted individual promotion of children’s competencies is sufficient to improve children’s skills, especially those preschoolers at heightened developmental risk. In this paper, we focus on attentional- and concentration-related developmental risks (compared to ADHD these risks are also developmentally inappropriate, but they do not have to necessarily persist over 6 months and are only focused on the preschool setting). Hence, if the state law is effective, age-adjusted improvements of DESK scores in this specific period of time would be expected.

Based on annual DESK survey waves, the research questions of this paper are as follows: (1) What is the proportion of children with a developmental risk at survey wave 1 (DESK-R-SW1, conducted in 2017), what is the proportion of the same children with a developmental risk at survey wave 2 (DESK-R-SW2, conducted in 2018)? Does the prevalence rate ratio improve over time? (2) Is the rate of improvements (i.e., developmental risk at survey wave 1, no developmental risk at survey wave 2) higher than the rate of deteriorations (i.e., no developmental risk at survey wave 1, but developmental risk at survey wave 2)?

## Method

### Study Region

MWP is a German rural state with a total population of 1.609 million (data for 2019) (Statistical Office of Mecklenburg-Western Pomerania Germany, 2019a). The economic and health-related profile of this region is rather challenging. The unemployment rate (7.1%) and the EU based at-risk-of-poverty rate (19.4%) are higher than the national average (5% and 15.8%, respectively) (Federal Employment Agency Germany, 2019). MWP preschools are attended by 94.9% of the 3- to 6-year-olds (national average: 93%) (Statistical Office of Mecklenburg-Western Pomerania Germany, 2019b).

### Study Design

The evaluation of the federal state law started in 2011 (*project name omitted for double blind reviewing*) including *n* = 100 participating “DESK-preschools.” Based on the total number of preschools in MWP in 2011, *n* = 1058 (Bock-Famulla & Lange, 2013), this reflects a participation rate of 9.4%. In 2018, *n* = 154 “DESK preschools” participated in the evaluation of the federal state law. Based on the total number of preschools in MWP in 2018, *n* = 1097 (Bock-Famulla et al., 2020), this represents a proportion of 14%.

### Instrument

To detect age-adjusted developmental risks among 3- to 6-year-olds in the domains of motor, linguistic, cognitive, and social-emotional skills the revised “Dortmunder Developmental Screening for Preschools (DESK 3–6 R)” was used (Tröster et al., 2016). Since the DESK version for 5- to 6-year-olds also aims to cover the learning preconditions for school entry, it includes the DESK domain “Attention and concentration” to assess impulse control, concentration, and sustained attention which are instrumental to later life functioning, e.g., in schools (Denham et al., 2014; Gestsdottir et al., 2014; Oriol et al., 2017). The items of this DESK domain are as follows:

- “Sets aside his/her own needs within the group.”
- “Waits for his/her turn.”
- “Occupies him- or herself with a task over a longer period of time.”
- “Continues performing an activity even if he or she gets distracted.”
- “Listens carefully to the preschool teacher’s explanations.”
- “Remains seated while eating, playing or doing handicrafts.”
- “Remembers agreements.”
- “Is aware of his or her own belongings.”

The DESK screening yields age-adjusted stanine (standard nine) values ranging between one and nine. Higher stanine values are associated with a more advanced level of development. A stanine value of one (corresponding to percentile ranks 0–4) indicates a developmental risk. In this case, an assessment, e.g., by a pediatrician is recommended. He/she evaluates whether or not this child needs further professional support (depending on the affected DESK domain, e.g., by a speech and language therapist, occupational therapist, or physiotherapist). A stanine value of two (corresponding to percentile ranks 5–11) denotes an inconclusive finding that does not allow a definite decision about the presence of a developmental risk. In this case, the screening has to be repeated at a later date. Stanine values from three to nine (corresponding to percentile ranks 12–100) indicate a normal development.

The tasks of the DESK domain “Attention and concentration” are documented based on routine observation by the regular preschool teachers. Referring to this DESK domain the inter-rater reliability was 0.29–0.63 (Cohen’s kappa coefficient; median kappa: 0.53) (Tröster et al., 2016). Cronbach’s alpha was *α* = 0.83. The DESK 3–6 has proven valid as a screening instrument (Tröster et al., 2011). Results in the DESK domain “Attention and concentration” are strongly associated with the two subscales “Attention” (*r* =  −0.61; *p* < 0.01) and “Hyperactivity-Impulsivity” (*r* =  −0.61; *p* < 0.01) of the “Diagnostic System for Children’s and Adolescents’ Mental Disorders” (Tröster et al., 2016) which are based on the ICD-10, and DSM-IV, respectively (Döpfner et al., 2008).

Since the DESK 3–6 was developed with and for practitioners this screening instrument is highly accepted by preschool teachers in Mecklenburg-Western Pomerania (Franze et al., 2010). Therefore, the DESK is likely to also be conducted in preschools which are not in the federal state law evaluation program.

In contrast to the Child Behavior Checklist CBCL (Arbeitsgruppe Deutsche Child Behavior, 2000) and the Strengths and Difficulties Questionnaire SDQ (Goodman, 1997), which are both more often used internationally, the DESK 3–6 R also aims to detect age-adjusted developmental risks in other developmental domains. Therefore, the main reason for choosing the DESK 3–6 R was to economically assess different developmental domains. The project as a whole is not only interested in attention and concentration, but also in other developmental domains. Another reason was that the DESK 3–6 R is an instrument developed with and for practitioners (compared with the SDQ and CBCL).

### Evaluation Data and Cases Included in the Analysis

**Fig. 1:**
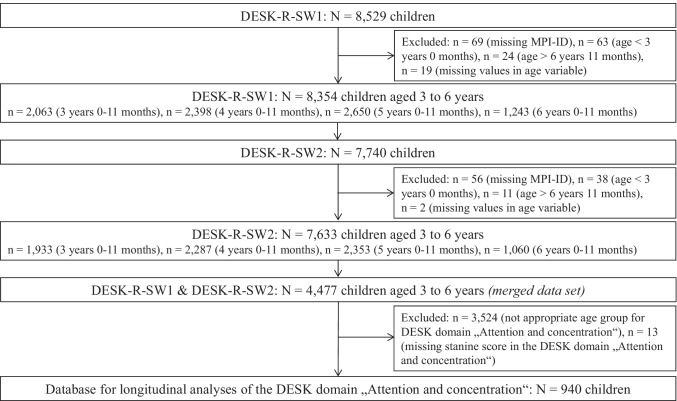
Consort diagram detailing the construction of the study population for the longitudinal assessment of age-adjusted DESK scores in the DESK domain “Attention and concentration” (*n* = 940)

DESK data from survey wave 1 (DESK-R-SW1, conducted in 2017) and survey wave 2 (DESK-R-SW2, conducted in 2018) were used. The longitudinal matching of DESK data was conducted using the ID Management solution E-PIX (Enterprise Identifier Cross Referencing). E-PIX allows for unambiguous participant management and efficient aggregation of research data (Bialke et al., 2015). E-PIX results in a child-specific Master Patient Index (MPI-ID) which is generated by using the variables surname, name, birth date, gender, and preschool ID. Missing values in these variables result in missing MPI-IDs. Children with missing MPI-IDs, missing DESK data, or missing stanine scores in the DESK domain “Attention and concentration” were excluded from the analysis (see Fig. 1).

### Implementation of the Study

Since May 2011, the Ministry for Health and Social Affairs MWP has annually provided the evaluation team with a list containing the names and locations of the specific “DESK-preschools.” Subsequently, the managing staff of each “DESK-preschool” has been contacted and informed about the DESK, the scientific evaluation, and measures to assure privacy protection. Parents are informed by means of an information letter that the participation of their child’s preschool in the DESK screening is mandatory but that it is possible to refuse permission for the completed DESK questionnaires to be passed on to the evaluation team (over the past years, however, only less than 5% have done so). Taking into consideration the Helsinki guidelines, this letter and a form for parental consent were developed by the evaluation team and sent to preschools. Both are delivered by the preschools and the form for parental consent is stored within the preschools (in the case of a parental refusal the DESK is still conducted by preschool teachers, but the completed DESK questionnaires are not shipped to the evaluation team).

Before conducting the DESK, the pedagogic preschool staff of each “DESK-preschool” participated in a training course to ensure standardization of the screening process. The training had previously been developed in a pilot project (Franze et al., 2010). Thus, trained preschool teachers conduct the DESK.

According to the contract between the Ministry of Social Affairs, Integration, and Gender Equality Mecklenburg-Western Pomerania and the evaluation team, no advice is given as to how to conceptualize the targeted individual promotion and there is no specific intervention for DESK preschools. Instead, annual meetings are offered by the evaluation team where “DESK preschools” can exchange their experiences.

The contents of the targeted individual promotion are assessed by means of written surveys addressed to the management staff of “DESK preschools.” Results from the survey conducted in 2018 reveal that the promotion of the DESK domain “Attention and concentration” consists of daily offerings implemented throughout the day within the daily routine. For example, in the morning circle preschoolers are encouraged to listen closely if someone speaks and not to interrupt each other. Further measures are concentration and memory games (e.g., “I pack my bags and take with me”), doing a jigsaw, story time, dialogic readings, table service, and yoga for children. Moreover, “DESK preschools” have also adopted specific programs to promote these skills, e.g., the valid and group-based “Marburger Konzentrationstraining” (Marburger concentration training) (Domsch et al., 2018; Krowatschek et al., 2010).

### Statistical Methods

The DESK scores 1 and 2 were summarized into one category “developmental risk/inconclusive finding.” The category “normal development” represents the DESK scores 3–9. We cross-tabulated the longitudinally assessed frequency of these two categories including the calculation of Fisher’s exact test. We then calculated the prevalence rate ratio (PRR). A PRR of 1 indicates no difference between the two survey waves. A PRR below 1 indicates that the proportion of children with “developmental risk/inconclusive finding” has decreased between DESK-R-SW 1 and 2. In this case, there is evidence for the effectiveness of the targeted individual promotion. This index helps to clarify whether there is a change in the likelihood that children have a “developmental risk/inconclusive finding” 1 year later (i.e., DESK-R-SW2). Since the PRR focuses on the effectiveness of the targeted individual promotion, which only considers children affected by a developmental risk in a survey wave, we further calculated the ratio of the rate of improvements (i.e., developmental risk/inconclusive finding at DESK-R-SW1, normal development at DESK-R-SW2) divided by the rate of deteriorations (i.e., normal development at DESK-R-SW1, developmental risk/inconclusive finding in DESK-R-SW2). A ratio > 1 indicates that the rate of improvements is higher than the rate of deteriorations. This index serves as an overall or net effect which illustrates not only the effect of the targeted individual promotion but also the effect of the promotion integrated into the daily routine which is targeted at all children (i.e., not only those previously affected by a developmental risk).

### Data Analysis

We calculated the DESK stanine scores by using the SAS statistical software package (Version 9.4, SAS Institute Inc., Cary, USA). To cross-tabulate we used IBM SPSS Statistics (Version 26, IBM, Armonk, USA). We calculated the PRR using STATA (Version 14.2, StataCorp, College Station, USA). To calculate the ratio of the rate of improvements divided by the rate of deteriorations, we used Microsoft Excel (Microsoft Office Professional Plus 2019, Version 1808, Redmond, Washington, USA). 

All inference statistics assume an α error probability of 0.05.

## Results

At DESK-R-SW1, 165 of 940 children were affected by a “developmental risk/inconclusive finding” in the DESK domain “Attention and concentration” (see Table 1). This equals a prevalence rate of 17.5%. At DESK-R-SW2, this proportion decreased to 128 out of 940 and thus a prevalence rate of 13.6%. Therefore, the PRR is 0.78. The decrease in the number of children affected by a developmental risk is statistically significant (*p* = 0.02).

**Table 1:** Categorized changes of results in the DESK domain “Attention and concentration” from DESK-R survey wave 1 (DESK-R-SW1) to DESK-R survey wave 2 (DESK-R-SW2)^1^ (*N* = 940)

Categorization of results		DESK-R-SW2				Total	Prevalence rate ratio	*p*	95% CI	Ratio of the rate of improvements^2^ divided by the rate of deteriorations^3^
		No finding		Developmental risk/inconclusive finding						
		*n*	*%*^1^	*n*	*%*^1^					
DESK-R-SW1	No finding	729	94.1	46	5.9	775				
	Developmental risk/inconclusive finding	83	50.3	82	49.7	165	0.78	0.02	[0.62, 0.95]	8.47
	Total	812		128		940				

*CI* confidence interval

^1^Percentage frequency based on results at DESK-R-SW2

^2^Improvements: risk at survey wave 1 (DESK-R-SW1), no risk at survey wave 2 (DESK-R-SW1)

^3^Deteriorations: no risk at DESK-R-SW1, risk in DESK-R-SW2

There is another interesting aspect worth highlighting: of the 165 children with “developmental risk/inconclusive finding” in the first survey wave, 1 year later (i.e., DESK-R-SW2) 83 children (50.3%) have a DESK result which is categorized as “no finding.” 82 children (49.7%) within this group are still affected by a “developmental risk inconclusive finding.” Of the 775 children’s DESK scores that were categorized as “no finding” at DESK-R-SW1, only 46 children (5.9%) were affected by a DESK result which was categorized as “developmental risk/inconclusive finding” 1 year later (i.e., DESK-R-SW2). For the remaining 729 children (94.1%), no change of the categorization “no finding” was observed. After 1 year, the proportion of the children with improved DESK scores was 8.47 times higher than the proportion of children whose DESK results had deteriorated.

## Discussion

The targeted individual promotion for children affected by a “developmental risk/inconclusive finding” at DESK-R-SW1 is effective as a considerable proportion of children improved their DESK scores leading to a screening result categorized as “no finding” in the consecutive survey wave 1 year later (i.e., DESK-R-SW2). This positive tendency can be explained by the detection of a developmental risk as a result of the previous DESK screening, which then led the preschool teachers to perform a targeted individual promotion. This result is remarkable, since the categorical change of the DESK result is based on a time period of just 1 year but, furthermore, on the chosen categorization (i.e., DESK scores 1 and 2 versus DESK scores 3–9). We presume that a developmental risk in this domain, e.g., because a child does not listen well enough to the preschool teacher’s explanations, becomes constantly visible in the preschool´s daily routine after conducting the DESK, e.g., during the daily meals or creative activities such as painting and handicrafts. This may lead to intensive use of promoting activities.

Regarding the effectiveness of the promotion integrated into the daily routine and targeted at all children (i.e., not only those affected by a developmental risk), the ratio of the rate of improvements divided by the rate of deteriorations indicates that there is a clear effect of the preschool’s promotion of the group as a whole. It is the intention of the legislation that the promotion of the children’s attention and concentration within the daily routine targets all children. Data from a questionnaire-based survey of the managing staff of the “DESK preschools” reveal that this promotion tends to consist of group activities, e.g., board games. Moreover, “DESK preschools” have also adopted specific programs to promote these skills, e.g., the “Marburger Konzentrationstraining” (Marburger concentration training). Therefore, these promoting activities seem to be in accordance with prevention research showing that the more intensive, the more comprehensive and the longer-lasting the early prevention, the more effective it is (Campbell & Ramey, 1994; Mayr, 2000). Nevertheless, it is important to point out that for 5.9% of the children a deterioration of the DESK result was observed. At DESK-R-SW1, these children were not affected by a developmental risk, but at DESK-R-SW2 their DESK results were categorized as “developmental risk/inconclusive finding.” This means that a positive DESK result can also change within a rather small-time period. This result is in line with current research findings. For example, Vella et al. longitudinally assessed the development of the scores of the SDQ (Vella et al., 2019) which includes the subscale “Hyperactivity” (Goodman, 1997) and is a good predictor for ADHD (Hall et al., 2019). The biannually assessed SDQ scores of 4- to 12-year-old children longitudinally revealed six different value patterns, i.e., a constantly low vs. high SDQ score (“Low Difficulty” (72.9%) vs. “High Difficulty” (2.2%)), a constant decrease vs. increase of mental health problems (“Improvers” (9.7%) vs. “Decliners” (7.9%)), a *U*-shape (“Early Improvers/Late Decliners” (2.7%)), and an inverted *U*-shape (“Early Decliners/Late Improvers” (4.7%)).

This further emphasizes the need to improve preschool promotion addressed to all children to decrease the prevalence rates of attentional- and concentration-related developmental risks.

### Limitations and Strengths of the Present Study

The limitations of our study are as follows:

1. There are a variety of variables used to assess the implementation of an intervention (e.g., dosage, fidelity, quality of delivery, participant responsiveness (Durlak, 2015)). Our study is limited to one assessment. Furthermore, the study does not include objective data on the pedagogical quality of the “DESK-preschools,” the children’s socioeconomic status, and DESK scores of a control group.
2. The DESK 3–6 R which was not primarily designed for research, but developed with and for practitioners is not a concentration test but indicates which children are at risk of attentional and concentration problems. Furthermore, the inter-rater reliability for the DESK domain “Attention and concentration” is only moderate. The results are therefore also dependent on the observer to a certain extent and are not entirely objective.
3. The contents of the targeted individual promotion reveal a broad scope of different interventions which leads to the open question what exactly worked.
4. In this analysis, we only analyzed the longitudinal data of two consecutive survey waves. The long-term effects of the federal state law remain unclear.

The strengths of our study are as follows:

1. As mentioned above, a low SES is a risk factor for educational and health outcomes. Therefore, early childhood education and care is especially important in social hotspots. Since our study was conducted in such hotspots, our analyses address a topic and a target group which is of high importance in terms of public health, social medicine, social epidemiology, and psychology.
2. Regarding the wishes of preschools to independently and individually plan their pedagogical promotion activities, the described federal state law has supported the decision to use a valid and standardized developmental screening instrument in all preschools involved in the evaluation. A consequence of the utilization of the same screening instrument is that “DESK preschools” can be compared, thus enabling the preschools to promote children’s competencies in an evidence-based way. This is worth mentioning since the prevalence rates of ADHD worldwide differ as a result of the use of different measuring instruments (Faraone et al., 2015).
3. Since “DESK preschools” have to conduct the DESK over a time period of at least 3 years, data analysis is not restricted to cross-sectional data. Instead, based on the use of E-PIX, we could unambiguously and efficiently match DESK scores at the level of the individual child, while still assuring a high level of data privacy.
4. The large number of “DESK preschools” involved, including preschools from all cities and administrative districts in MWP, leads to evaluation data that is representative for low SES regions in this federal state. Furthermore, this extended database allows for subgroup analyses allowing well-defined inclusion criteria and still maintaining sufficient statistical power.

### Future Prospects

The DESK data should be complemented by data related to the parents, e.g., their parenting style and their mental health. Parenting can serve as a contributing factor in social skills, early literacy, and later psychological health (Barlow et al., 2014; Hindman & Morrison, 2012). For example, a “directive parenting style” can affect literacy because it rarely encourages children’s speech development (von Suchodoletz, 2007). Furthermore, children’s mental health problems can significantly increase if their parents suffer from mental illness and/or substance abuse (Bosanac et al., 2003; Klein, 2001; Rasic et al., 2014; Riley et al., 2008).

The evaluation of this federal state law also includes data from the school entry examination (SEE). To contribute to a further standardization of the SEE, the Ministry of Economics, Employment and Health MWP invited the evaluation team to participate in several management meetings of the public health officers in MWP. In this context, the evaluation team recommended the application of the German version of the SDQ (Woerner et al., 2002) in school entry examinations in all cities and administrative districts in MWP. This recommendation was positively received and pilot-tested in selected administrative districts. Since the association of the DESK with the SDQ has only been calculated for 3- to 4-year-olds, we can now analyze the associations of DESK scores for 5- to 6-year-olds (especially those in the DESK domain “Attention and concentration”) and SDQ scores to further validate the predictive value of the DESK for the results of the school entry examination. Moreover, these analyses are useful to continuously monitor the effectiveness of this federal state law, especially regarding its aim to reduce social inequalities.

In order to be able to answer the question of which promotion activity work and how, future analyzes could be more restrictive and therefore carried out taking inclusion criteria into account (e.g., only preschools using the “Marburger concentration training”). Analyzes on the effects could then concentrate on different implementation levels of the preschools included and also consider the hierarchical structure of data (i.e., children nested within preschool groups nested within preschools).

Since we only used data from a 1-year follow-up, further research is needed to estimate the long-term effects of this federal state law. However, the DESK domain “Attention and concentration” is only applicable for 5- to 6-year-olds. Therefore, further data are needed, especially assessments of primary school-aged children. The impact of a low SES on ADHD and the impact of ADHD on children’s health and educational status mean that it is important that there is continuity with the promotion of children’s attention and concentration being continued in primary schools. Primary schools could use evidence-based DESK results previously collected by preschools to plan ongoing promoting activities at the evidence-based level.

## Conclusions

The results provide evidence for the effectiveness of 1-year individual and group level promotion in preschools referring to the DESK domain “Attention and concentration.” Nevertheless, over the same time period, the DESK results of some children are deteriorating. Therefore, preschools have to be aware of the natural course of events over time leading some children to show modest declines in their skills.
